# Molecular Epidemiology of Sporadic and Outbreak-Related *Salmonella* Typhi Isolates in the Brazilian North Region: A Retrospective Analysis from 1995 to 2013

**DOI:** 10.3390/idr14040060

**Published:** 2022-08-03

**Authors:** Ana Judith Pires Garcia Quaresma, Yan Corrêa Rodrigues, Joseline Barbosa Aboim, Mayza Miranda Bezerra, Maria Isabel Montoril Gouveia, Ana Roberta Fusco Da Costa, Cintya de Oliveira Souza, Flávia Corrêa Bastos, Luana Nepomuceno Gondim Costa Lima, Francisco Lúzio de Paula Ramos, Karla Valéria Batista Lima

**Affiliations:** 1 Bacteriology and Mycology Section, Evandro Chagas Institute (SABMI/IEC), Health Surveillance Secretariat, Ministry of Health, Ananindeua 67030-000, PA, Brazil; anaquaresma@iec.gov.br (A.J.P.G.Q.); yan.13@hotmail.com (Y.C.R.); line_barbosaa@yahoo.com.br (J.B.A.); mayzamiranda9@gmail.com (M.M.B.); isabelmontoril13@gmail.com (M.I.M.G.); anacosta@iec.gov.br (A.R.F.D.C.); cintyaoliveira@iec.gov.br (C.d.O.S.); flavia_bastos@hotmail.com (F.C.B.); luanalima@iec.gov.br (L.N.G.C.L.); 2 Ph.D. Program in Parasitic Biology in the Amazon Region (PPGBPA), State University of Pará (UEPA), Tv. Perebebuí, 2623-Marco, Belém 66087-662, PA, Brazil; 3 Evandro Chagas Institute (SABMI/IEC), Health Surveillance Secretariat, Ministry of Health, Ananindeua 67030-000, PA, Brazil; franciscoluzio@iec.gov.br

**Keywords:** *Salmonella* Typhi, laboratory surveillance, genotyping

## Abstract

Typhoidal salmonellosis is a global public health problem occurring in developing endemic regions. In Brazil, cases are mostly registered in the North and Northeast regions. Molecular characterization of the strains is important to understand the epidemiology of disease infections and to design control strategies. The present study retrospectively evaluates the genotyping features of sporadic and outbreak-related *Salmonella* Typhi isolates from the Brazilian North region. Bacterial isolates were recovered from blood and a rectal swab of patients in the states of Acre and Pará, Brazilian North region, in the period of 1995 to 2013, and were submitted to genotyping by applying Multilocus sequence typing (MLST) and Pulsed Field Gel Electrophoresis (PFGE) reference methods. MLST genotyping revealed the presence of epidemic clones ST1 and ST2, and 20 pulsotypes were identified by PFGE, including four distinct clusters (A–D), and six subclusters (A1–D1) with indistinguishable strains in different periods and locations. To conclude, the obtained data demonstrates the temporal stability, adaptation, and transmission of outbreak-related and sporadic *S.* Typhi strains over time, contributing to the transmission chain in the region.

## 1. Introduction

Typhoidal salmonellosis is a global public health problem associated with deficient socioeconomic levels in developing areas of Africa, the Americas, South-East Asia, and the Western Pacific regions, reaching a burden of 11–20 million cases annually, resulting in about 128,000–161,000 deaths per year [1]. In Brazil, typhoid fever occurs in an endemic form, particularly in the North and Northeast regions, which accounted for over 88.0% of confirmed cases in the period 2010–2019, reflecting the living conditions of their populations [2].

*Salmonella* Typhi, the etiologic agent of typhoid fever, causes a high fever and severe gastrointestinal issues, including life-threatening intestinal perforations, and is usually spread via contaminated food and water [3,4,5,6]. In contrast to other *Salmonella* species, *S.* Typhi is man-restricted [7].

The epidemiological investigation supported by molecular methods for *S.* Typhi is important for disease control, such as during a disease outbreak, to trace the potential sources. Therefore, the present study proposed to retrospectively evaluate the genetic relatedness of sporadic and outbreak-related *S.* Typhi isolates from two states in the Brazilian North region in the period of 1995 to 2013. 

## 2. Materials and Methods

### 2.1. Bacterial Isolates and Identification

Thirty-eight *S.* Typhi isolates collected in the Bacteriology and Mycology Section of the Evandro Chagas Institute (IEC), Ananindeua, Pará, Brazil, were evaluated. The IEC is a regional referral center for typhoid fever diagnosis, and one of the few centers in Brazil, and perhaps in Latin America, that maintains a clinical and laboratory surveillance program for typhoid fever, operating since the 1980s in the investigation of sporadic cases and outbreaks that occurred in different locations in the region. 

Bacterial isolates were recovered from blood and rectal swabs from patients in the states of Acre and Pará, Brazilian North region, in the period of 1995 to 2013. Isolates were reactivated to confirm identification, viability, and purity by the first growth in TSB broth (Tryptic Soy Broth) at 35 °C for 24 h, followed by plating on salmonella shigella agar (SSA) at 35 °C for 24 h. Colonies that did not ferment lactose, characteristic of *S.* Typhi, were selected and inoculated in TSI (Triple Sugar Iron) medium to the definition of the biochemical profile, then incubated at 37 °C for 24 h. Subsequently, they were directed to biochemical identification using the VITEK-2 automated system, according to the manufacturer’s instructions.

### 2.2. Molecular Typing by Multilocus Sequence Typing–MLST

MLST was performed as previously described by Harbottle et al. [8], with modifications. The seven housekeeping genes in the protocol (*aroC*, *dnaN*, *hemD*, *hisD*, *purE*, *sucA* and *thrA*) were amplified by PCR on the Veriti thermocycler (Applied Biosystems, Foster City, CA, USA). PCR products were separated by electrophoresis in 1.5% agarose 1X TAE gel stained with SYBR Safe (Invitrogen) and bidirectionally sequenced using Big Dye Terminator v3.1 chemistry on an ABI Prism 3130 Genetic Analyzer (Applied Biosystems, Foster City, CA, USA). The results obtained were analyzed according to the PubMLST database (https://pubmlst.org/organisms/salmonella-spp/, accessed on 14 March 2022) for the determination of sequence types (STs).

### 2.3. Molecular Typing by Pulsed-Field Gel Electrophoresis-PFGE

PFGE was performed according to the Gautom [9], with slight modifications. The genomic DNA of *S.* Typhi isolates was digested using restriction endonuclease XbaI enzyme for 24 h. An electrophoretic run was performed using the CHEF-DR III (Bio-Rad) system for 24 h at 160 V, followed by band patterns analysis and dendrogram construction applying the Dice coefficient with 3% tolerance, 80% cut-off, and UPGMA using the software BioNumerics 6.5 (Applied Maths).

## 3. Genotyping Results

**Figure 1:**
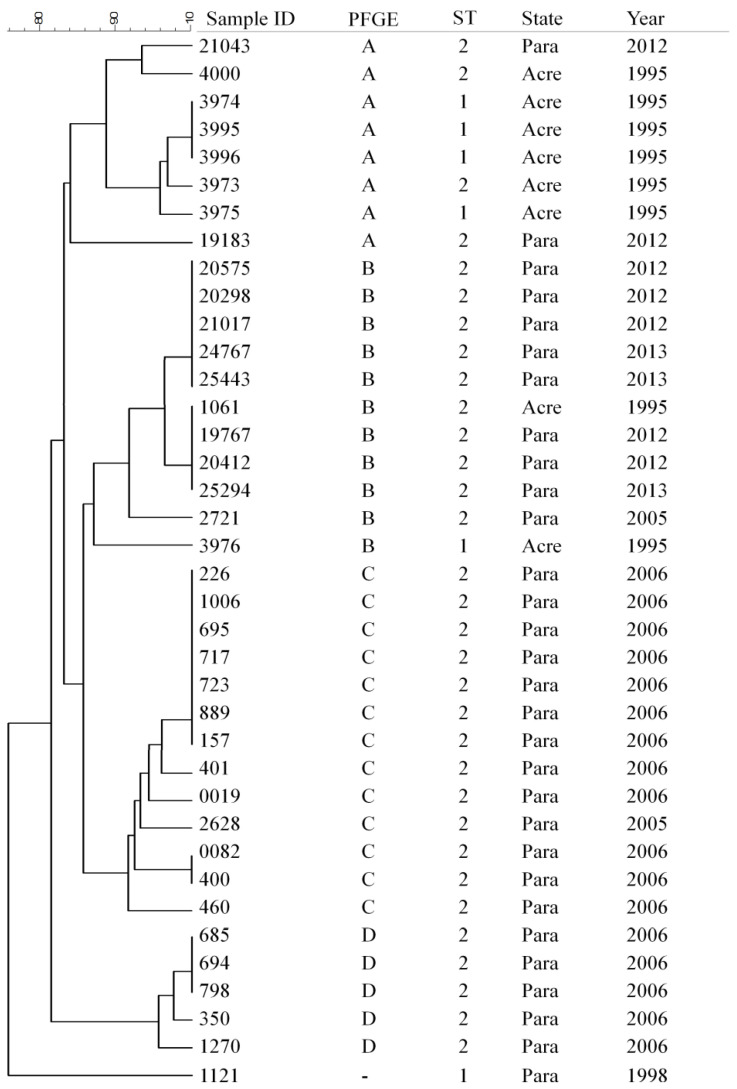
Dendrogram based on PFGE analysis demonstrating the genetic relatedness of 38 *S.* Typhi isolates from Acre and Pará States, Brazilian Amazon, in the period of 1995 to 2013.

MLST genotyping revealed the presence of epidemic clones ST2 (32/84.2%) and ST1 (6/15.8%). Through the PFGE analysis, 20 pulsotypes were identified, including four distinct clusters (A–D), six subclusters (A1–D1) with indistinguishable strains, and one unrelated strain (Figure 1). *S.* Typhi strains with similar genetic signatures by PFGE were detected in distinct periods and locations: Strains in cluster A were demonstrated to be genetically related with isolates from 1995–2012 in Acre and Pará States, also being associated with epidemic clones ST1 and ST2; ST1 strains were mostly detected at Acre State in 1995, including three clonally related isolated as verified on subcluster A1. In contrast, ST2 strains were predominant in cluster B, including subclusters B1 with strains from Pará in 2012–2013 and subclusters B2 with strains from Acre and Pará in 1995, 2012, and 2013. Finally, clusters C and D were mostly composed of ST2 strains from 2006 in Pará State.

## 4. Discussion

The Brazilian North region is an endemic area for typhoid fever, especially the state of Pará, which has historically registered cases and outbreaks of typhoid fever since the 1980s. The identification of circulating clones and understanding of transmission by applying reference molecular epidemiology tools, such as PFGE and MLST, are of high value to control strategies and the comprehension of transmission dynamics in this region. Through a retrospective approach, the present report provides data on the molecular epidemiology of sporadic cases and outbreak-related *S.* Typhi strains in the Brazilian North/Amazon region from 1995 to 2013. 

The most globally spread MLST lineages ST1 and ST2 were the only detected clones in the analysis period and locations, demonstrating a low genetic diversity of MLST clones in the region, likewise reported in Bangladesh, Angola, Zambia, India, Malaysia, Chile, and Papua New Guinea [10,11,12,13,14,15]. It is important to highlight that most of the studies applying genotyping methods are based on a retrospective approach, evaluating isolates since the 1980s. Also, this is the first report demonstrating the restricted presence of epidemic clones ST1 and ST2 in the Brazilian North region since 1995 and confirms the predominance of ST1 and ST2 co-existing in developing endemic regions. 

The PFGE typing revealed a higher genetic diversity of *S.* Typhi strains, with the presence of 20 distinct pulsotypes, and clonally related isolates causing infection in different locations and periods, also supporting the hypothesis of a localized and restricted clonal expansion [10,11]. Such results are different from those reported by Tiba-Casas et al. [16] evaluating isolates from São Paulo, Brazil, which described a genetic background mostly composed of two main clonal clusters. Interestingly, strains ST1 and ST2, belonging to clusters A and B from Acre/1995, were related to an outbreak that occurred in the city of Tarauacá/Acre State and was detected after 17 years causing infection in the city of Belém/Pará State (subclusters A1 and B2). Strains 1006, 695, 717, 723, 889, 401, 0082, 400, and 460, included in cluster C, were related to an outbreak that occurred in 2006 in the Guamá neighborhood, Belém/Pará; the other strains from the same period and cluster were from epidemiologically unrelated sporadic cases, even though such strains present a certain level of genetic relatedness. The strains belonging to clusters B and D, recovered in 2006, 2012, and 2013, were from epidemiologically unrelated sporadic cases. 

The present data demonstrate the temporal stability, adaptation, and transmission of outbreak-related and sporadic *S.* Typhi strains over time and suggest the role of asymptomatic human hosts, who usually develop chronic infection, eliminating the pathogen through feces that contaminate water and food and contribute to the transmission chain in the region. Finally, further studies evaluating *S.* Typhi isolates from recent periods allow comparison with the data present in this report, in addition to the study of antimicrobial resistance patterns, virulence features, and genetic diversity that are of high value to the comprehension of epidemiology and dynamics of typhoidal salmonellosis in this endemic region.

## Data Availability

All relevant data is presented within the manuscript.
